# Radium in the Treatment of Deafness

**Published:** 1914-04-18

**Authors:** Howard S. Willson

**Affiliations:** Weybridge


*To the Editor of* The Hospital.

### Radium in the Treatment of Deafness.

*To the Editor of* The Hospital.

Sir,—In your issue of March 28 you published a letter from Dr. H. D. McCulloch on the subject of "Radium in the Treatment of Deafness." I am not qualified to express any general opinion as to the value of radium and ultra *x*-rays in cases of deafness, but I should like to call your attention to a particular case of deaf-mutism (apparently "acquired") in a little girl who was a patient of mine for some five years (1906-12).

I enclose notes of her case, taken from a paper read by Dr. McCulloch before the British Oto-Laryngological Society in October last; and I can confirm all that he says there in regard to the improvement of the child's speech and hearing after a prolonged course of treatment with ultra *x*-rays. "One swallow does not make a summer," and as I have already stated, I do not myself express any opinion as to the general value of Dr. McCulloch's treatment. I merely wish, in common fairness to him, to call attention to this particular case for what it is worth.—I am, Sir, yours faithfully,

Weybridge, April 5. Howard S. Willson.

[This appears to refer to one of the cases instanced by Dr. McCulloch. We are pleased to hear of the improvement, and thank our correspondent for his fair and temperate remarks.—Ed., The Hospital.]

